# Supplementary material for: Are carbon emissions trading and green financial instruments synergistic? -Comprehensive quantitative research based on content analysis
Source: PLoS One. 2024 Mar 7;19(3):e0298601. doi: 10.1371/journal.pone.0298601 (PMC10919601; doi:10.1371/journal.pone.0298601)
Supplement: S1 Data — (ZIP) [file pone.0298601.s001.zip › Data/地级市Z_F工作报告.zip!/╔╜╢1⁄2/┴─│╟╩╨/└┤╫╘╣1⁄2╓┌║┼í╛┬φ┐╦╩2╛▌═°í┐_2016.docx]

各位代表：

　　现在，我代表市人民政府向大会报告工作，请予审议，并请市政协各位委员提出意见。

　　一、“十二五”时期政府工作回顾

　　“十二五”时期是我市发展历程中极不平凡的五年。五年来，面对国内外环境复杂变化和诸多挑战，全市上下在省委省政府和市委的坚强领导下，抢抓发展机遇，积极应对挑战，全力推动科学发展、跨越发展，全市经济、政治、文化、社会、生态文明建设都取得巨大成就，“十二五”规划确定的主要目标任务胜利完成。

　　（一）综合实力迈上新的台阶。面对国际金融危机持续影响和国内经济下行压力，全市经济保持平稳较快发展，主要指标增幅高于全省平均水平。2015年，全市生产总值完成2663.6亿元，是2010年的1.7倍，年均增长10.6%，人均达到7185美元；地方一般公共预算收入175.9亿元，是2010年的2.5倍，年均增长20.1%；社会消费品零售总额1060.2亿元，是2010年的2倍，年均增长14.6%；本外币各项存款余额2592.3亿元，贷款余额1872.3亿元，分别是2010年的2.1倍和1.9倍。

　　（二）转型升级取得重大进展。全市三次产业比例调整为11.9∶51∶37.1，第三产业占比提高7.7个百分点。农业结构调整深入推进。粮食生产实现“十三连增”，达到“吨粮市”标准，被评为全国粮食生产先进单位。瓜菜菌总产达到1626万吨，被评为“中国蔬菜第一市”。规模以上龙头企业主营业务收入、“三品”认证数量分别是2010年的2.4倍和7.7倍，农业机械化率提高11个百分点。工业经济扩量提质。规模以上工业企业达到2867家，主营业务收入突破9000亿元，年均增长17.5%。传统产业改造、新兴产业培育、工业园区建设取得新成效，高新技术产业占比达到26.3%。服务业发展步伐加快。全市旅游总收入年均增长19.3%，社会物流总额突破1万亿元，电商企业达到3600家，被命名为“中国温泉之城”。科技创新和节能减排成效明显。全市新增国家级研发平台4家、省级79家，荣获中国专利金奖1项、全国质量奖和省长质量奖4项，成功创建国家环境保护模范城市，代表山东省参加全国海河流域水污染防治检查荣获第一名。

　　（三）发展后劲得到显著增强。全社会固定资产投资累计完成7747亿元，年均增长18.8%。招商引资到位资金3350亿元，是“十一五”的3.7倍。信发集团6×66万千瓦机组、鲁西化工新材料、中通新能源客车、中色奥博特铜精深加工等一批重大项目建成投产。基础设施建设实现突破。全市电网投资完成69.4亿元，是“十一五”的2.7倍。新建改建公路6732公里。京九铁路聊城站房改造、邯济铁路扩能改造、高邢高速、德商高速聊城至范县段、济聊一级公路聊城段全面建成，青兰高速、莘县至南乐高速开工建设，郑济高铁、京九高铁、聊泰铁路、聊城机场、德郓高速等全部列入国家和省综合交通网规划，聊城畅通高效的现代交通体系正在形成。

　　（四）城乡面貌发生巨大变化。全市住房和城乡建设累计完成投资2970亿元，是“十一五”的10倍；常住人口城镇化率达到46.2%，提高10.1个百分点。城市规模明显扩大。中心城区由69平方公里扩展到90平方公里。全市达到292平方公里，是2010年的1.4倍。城市改造明显加快。棚户区改造征收面积2823万平方米，是“十一五”的5.6倍；开工建设回迁安置房10.7万套、基本建成10.4万套，5.4万户群众乔迁新居。城市功能明显增强。全市新建改建城市道路500公里、雨污管网675公里，增加新能源公交车1200部、供热管道200公里、管道天然气用户19.8万户。新建污水处理厂16个、垃圾处理厂9个，污水处理率和生活垃圾无害化处理率分别达到95%和100%。城市形象明显提升。中华水上古城风貌基本形成，徒骇河滨河大道、湖南路徒骇河大桥建成通车，市体育公园、市民文化活动中心等投入使用。各县（市）城区新建湖泊7个、湿地23个，水城特色进一步凸显。城乡环境明显改善。全市林木绿化率达到37.5%，提高6.3个百分点。中心城区新增提升绿化面积3500公顷，顺利通过国家卫生城市复审。小城镇建设步伐加快，建设农村新型社区510个，城乡环卫一体化实现全覆盖，城乡面貌发生显著变化。

　　（五）发展活力有了明显提升。农业农村改革扎实推进，土地确权登记颁证基本完成，农民专业合作社实现“一村一社”全覆盖。国企改革继续深化，国资监管体系更加完善。投融资体制改革和营商环境建设成效显著，设立了市级股权引导基金，市场主体达到30.1万户，是2010年的2.5倍。金融改革成果丰硕，新增市级银行业金融机构8家，农信社银行化改革全面完成；高唐天泰集团成功在英国上市，16家企业在“新三板”挂牌，总量居全省第7位。医疗卫生体制改革更加深入，东阿公立医院综合改革走在全国前列。对外开放取得重要进展，累计利用外资5.2亿美元，连续三年入围全国外贸百强市，境外投资企业达到91家。新设立了高新技术产业开发区、江北水城旅游度假区，经济技术开发区进入国家级开发区行列。

　　（六）群众生活实现大幅改善。全市财政用于民生的支出达到1028.9亿元，占总支出的80.3%，高于全省3.9个百分点。城镇和农村居民人均可支配收入年均分别增长11.4%和13.9%，57.6万人实现脱贫。就业创业成效显著。新增城镇就业35.6万人、农村劳动力转移就业45.3万人，城镇登记失业率控制在3.3%以内。社保体系更加完善。连续4年提高居民基础养老金，企业退休人员基本养老金、城乡居民医保财政补助、失业保险、城乡低保、农村五保等标准逐年提高，城乡医保制度实现整合，基本社保覆盖率达到87.5%。全市新建农村“幸福院”338处，新增社会养老床位1.5万张，市儿童福利院投入使用，慈善捐款达到3.4亿元。建设各类保障房4.4万套，改造农村危房5.3万户。教育事业健康发展。建成乡镇中心幼儿园135所，新建、改建中心城区中小学15处、农村校舍160万平方米，聊城市技师学院、聊城高级财经职业学校投入使用。卫生计生水平明显提高。市人民医院成为省级区域医疗中心，县乡医疗机构全部实行“先诊疗后付费”，低生育水平保持稳定。文化体育事业不断进步。新建改造综合性文化服务中心1000个，市豫剧院投入使用，成功举办了市第二届运动会。社会治理能力全面提升。法治聊城、平安聊城建设深入推进，安全生产事故指标全面下降。国防动员工作扎实开展，成功实现全国双拥创城“三连冠”。新闻出版、广播电视、社会科学、民族宗教、档案史志、台湾事务、防震减灾、妇女儿童、青少年、油区、气象等工作都取得新的成绩。

　　（七）自身建设迈出坚实步伐。深入开展党的群众路线教育实践活动、“三严三实”专题教育，严格落实“八项规定”，切实加强自身建设。政府职能加快转变。政府机构改革全面完成，事业单位分类改革扎实推进。在全省率先推出“三个清单”，推行“三证合一”、“四证联办”、“先照后证”，开展国地税联合办税，实行了星期六企业工作日、群众工作日和金融工作日制度，建立了网络信息办公系统。依法行政全面推进。出台了政府议事规则和重大行政决策程序规定，建立了法律顾问制度，完善了新闻发言人制度，制定了公共资金使用透明化等四项制度，建设了公共资源交易统一平台，对市长公开电话系统进行了升级改造。自觉接受各方面监督，办理人大议案和代表建议586件、政协委员提案935件。党风廉政建设成效显著。查处违反中央“八项规定”及“庸懒散”等问题1285起，给予党政纪处分778人，正风肃纪取得重要成效。

　　各位代表，五年来，聊城不仅经济社会发展取得巨大成就，而且被列入中原经济区、京津冀协同发展区、省会城市群经济圈和山东西部经济隆起带，成为国家和省区域发展战略叠加区，被评为国家现代农业示范区、国家节能减排财政政策综合示范城市、国家质量强市示范城市、国家循环经济示范城市。这为聊城今后长远发展提供了新的重大机遇。能够取得这样的成绩确实不易。这是中央、省委省政府和市委科学决策、正确领导的结果，是市人大、市政协和社会各界加强监督、大力支持的结果，是全市上下齐心协力、奋力拼搏的结果。在此，我代表市人民政府，向全市人民，向各位人大代表和政协委员、离退休老同志，向各民主党派、工商联、人民团体和各界朋友，向驻聊部队官兵和中央、省驻聊各单位，向所有关心支持聊城发展的海内外朋友，致以崇高的敬意和衷心的感谢！

　　各位代表，回顾五年来的工作，我们深切感到，要牢固树立以人民为中心的发展理念，切实做好政府工作，必须政治坚定、服务大局，坚决贯彻落实中央、省委省政府和市委的各项决策部署，紧密结合聊城实际，创造性地开展工作。必须解放思想、抢抓机遇，用心谋划、主动有为，努力把聊城的发展融入全国、全省发展的总体布局。必须改革创新、攻坚克难，敢于担当，勇于负责，积极破解发展难题，开创各项工作的新局面。必须争先进位、科学发展，坚持高起点、高标准，加快转方式、调结构，努力在全国、全省走在前列。必须凝心聚力、团结实干，调动好各级各方面特别是广大企业家的积极性，营造干事创业的浓厚氛围。必须作风引领、为民务实，切实强化服务意识，保持同广大群众的血肉联系，筑牢改革发展的坚实基础。这既是过去五年经验的总结，也是今后政府工作的重要遵循。

　　各位代表，在看到成绩的同时，我们也清醒地认识到，我市在发展中仍然存在一些问题和不足，主要是：经济总量偏小，人均占有水平较低；农产品深加工和品牌建设薄弱，农业产业组织化程度低；重化工业比重高，服务业发展滞后；科技创新能力薄弱，高新技术产业占比较低；经济外向度不高，利用两个市场、两种资源的能力不强；城镇化水平低，城市管理和公共服务体系还不完善；节能减排压力较大，大气污染防治任务艰巨；农村贫困人口较多，安全生产、社会稳定仍然存有隐患；政府职能转变还不到位，营商环境仍不够优化。对这些问题，我们一定高度重视，下决心予以解决。

　　二、“十三五”经济社会发展的主要目标和重点任务

　　各位代表，“十三五”时期，是全面建成小康社会的决胜期、全面深化改革的攻坚期，也是我市科学发展、跨越发展的关键期，制定并实施好“十三五”规划，具有重要的现实意义和深远的历史意义。根据市委十二届九次全会关于制定“十三五”规划的建议，我们制定了《聊城市国民经济和社会发展第十三个五年规划纲要（草案）》，已印发大会，请一并予以审议。

　　“十三五”时期，我们要全面贯彻党的十八大和十八届三中、四中、五中全会精神，深入贯彻落实习近平总书记系列重要讲话和视察山东重要讲话、重要批示精神，协调推进“五位一体”总体布局和“四个全面”战略布局，认真践行创新、协调、绿色、开放、共享五大发展理念，坚持发展第一要务，以提高质量和效益为中心，同步推进新型工业化、城镇化、信息化、农业现代化和绿色化，构建富有生机活力的开放型经济体系，将聊城建设成为冀鲁豫三省交界综合交通枢纽城市、经济社会发展新高地和中原经济区区域中心城市、京津冀协同发展试验区、中国北方高品质示范城市，确保与全省同步全面建成小康社会。主要发展目标是：

　　——综合经济实力显著增强。主要经济指标增幅高于全省平均水平，人均生产总值达到全国平均水平。全市生产总值年均增长8%左右，人均突破1万美元。地方一般公共预算收入年均增长8%左右，固定资产投资年均增长13%左右，社会消费品零售总额年均增长10%左右。

　　——现代产业体系基本形成。农业做优、工业做强、服务业做大，三次产业比例大体调整为8∶45∶47，产业竞争力显著提升。

　　——城乡区域发展更加协调。“聊茌东”都市区格局基本形成，县（市）城区功能更加完善，小城镇特色更为突出，新农村建设扎实推进，常住和户籍人口城镇化率分别达到56%和45%以上。

　　——生态环境质量明显改善。建成国家节能减排财政政策综合示范城市和国家循环经济示范城市，水和大气质量显著改善，打造河湖秀美、宜居宜业的良好环境。

　　——人民生活水平普遍提高。法治聊城、平安聊城、健康聊城、智慧聊城、幸福聊城建设取得重大进展，城镇和农村居民人均可支配收入年均分别增长8%和9%以上，全面打赢脱贫攻坚战。

　　——社会文明程度全面提升。社会主义核心价值观更加深入人心，优秀传统文化得到深入挖掘和阐发，建成覆盖城乡的公共文化服务体系，成为具有浓郁地方特色和较强竞争力的文化名市。

　　——各项体制机制更加完善。重点领域和关键环节改革持续深化，政府与市场、社会的关系进一步理顺，社会治理体系和治理能力现代化明显提升。

　　各位代表，实现“十三五”规划目标，必须主动适应新常态、把握新常态、引领新常态，切实把五大发展理念和依法治国要求贯穿经济社会发展全过程。

　　（一）突出创新发展，建设品质活力新聊城。把创新摆在发展全局的核心位置，以改革为动力，推进各领域跨界融合，构建富有活力、结构优化、高效协同的经济运行体系。到2020年，全社会研发投入占生产总值比重达到2.5%左右，发明专利申请量年均增长10%以上，高新技术产业占比达到35%以上，争创国家级高新技术产业开发区，全面建成国家现代农业示范区、国家质量强市示范城市，形成科技、经济、社会协调发展新格局。

　　（二）突出协调发展，建设融合跨越新聊城。推进区域协调，积极融入“一带一路”等国家和省区域发展战略。推进城乡一体，构建以“聊茌东”都市区为龙头、县（市）城区和中心镇为主体、美丽乡村为基础的新型城镇化格局，“聊茌东”城镇人口达到200万人，其他5个县（市）城区人口均达到25万人以上,城中村改造全面完成。推进重大基础设施建设，郑济高铁、德商高速、青兰高速、莘县至南乐高速建成通车，开工建设京九高铁、聊泰铁路、德郓高速、聊城机场，构建“米字型”铁路网、“田字型”高速公路网，实现县县通高速，建成冀鲁豫三省交界综合交通枢纽城市。

　　（三）突出绿色发展，建设秀美宜居新聊城。建设全域水城，实施市县水系连通工程，打造河湖秀美大水城。全面加强生态建设，实施道路、城镇、村庄、水网绿化工程，加快森林公园建设，建成国家森林城市。到2020年，全市空气质量基本达到国家二级标准；重要水功能区水质达标率达到86.8%，城市集中式饮用水源地主要指标全面达标，形成天蓝地绿水清、空气清新怡人的良好环境。

　　（四）突出开放发展，建设包容大度新聊城。坚持对内对外开放并重，加大市外境外招商引资、招才引智力度，着力引进世界500强、中国500强和行业50强，创新国际贸易发展方式，加快建设“海外聊城”，在引进全球优秀创业创新人才和团队、招大引强、重点企业国际化上实现突破，进出口总额年均增长6%左右。

　　（五）突出共享发展，建设和谐幸福新聊城。实施扶贫攻坚三年行动计划，实现399个省级重点贫困村整体脱贫。全市城镇新增就业21万人以上，登记失业率控制在4%以内。着力建设教育强市和人力资源强市，加快普及学前教育，推进义务教育均衡发展，提高普通高中办学质量，全面提升职业教育、高等教育和特殊教育水平。推进“健康聊城”建设，完善医疗卫生服务体系，争创国家食品安全城市；城市社区建成15分钟健身圈，农村普及便捷实用的体育健身设施。实施全民参保登记计划，社会保险基本实现法定人员全覆盖，社会福利救助体系更加完善，构建起覆盖全社会更均等、更公平、更高层次的保障网。

　　（六）突出依法治市，建设文明诚信新聊城。推进法治聊城建设，完善基层民主和群众自治制度，提高依法行政水平，促进基本公共法律服务均等化、便民化。推进精神文明建设，健全科技馆、少年宫等公共文化设施，提升市民文明素质，创建全国文明城市。推进诚信聊城建设，构建“守信受益、失信受损”的信用环境。推进平安聊城建设，建立网格化社会治安管理体系；完善安全生产管理体制，开展企业法人等普法教育，有效遏制重特大安全生产事故发生。到2020年，全民法律素养、城乡文明程度、社会诚信度、居民安全感大幅提高，我市成为依法办事、安全稳定、近悦远来的文明诚信之城。

　　各位代表，通过五年的努力，我市将实现综合实力大幅提升，产业体系迈向高端，交通出行方便快捷，社会环境安定和谐，水城特色更加突出，生态环境清新怡人，人民群众殷实幸福，一个文明、富裕、开放、秀美的新聊城将在鲁西大地蓬勃崛起！

　　三、2016年的目标任务和工作重点

　　2016年是“十三五”的开局之年，也是推进结构性改革的攻坚之年。根据中央、省委省政府和市委的决策部署，我们要坚持稳中求进工作总基调，按照“作风引领、团结实干、争先进位、科学发展”的总体要求和“123451”的发展思路，加快供给侧结构性改革和体制机制创新，积极落实“三去一降一补”五大重点任务，努力实现经济社会发展新突破。主要预期目标是：生产总值增长8%，地方一般公共预算收入增长8.6%，固定资产投资增长13%，社会消费品零售总额增长10%，实际利用外资增长10%，进出口总额增长6%，城镇居民人均可支配收入增长8%以上，农村居民人均可支配收入增长10%以上，居民消费价格涨幅控制在2%左右，全面完成省下达的各类约束性指标。工作中，重点抓好以下七个方面：

　　（一）着力推进转型升级，确保产业素质实现新提升。坚持以标准、质量、品牌培植核心竞争力，以管理、工艺、技改提质增效，推动三次产业又好又快发展。

　　推进农业发展方式转变。以建设国家现代农业示范区为抓手，发挥优势，补齐短板，努力提高组织化、产业化水平，加快向生态安全、优质高效方向发展。一是加快构建现代农业产业体系。以发展精致农业为重点，积极调整农业品种结构，大力发展精深加工，促进一二三产业融合，重点抓好20个精致农业园区和100个品牌蔬菜、特色林果、现代畜牧和水产养殖基地建设，实施好“百万头肉驴养殖工程”；建设乡村旅游示范点100个、创意农业基地10处。二是加快构建现代农业生产体系。以培育聊城区域公共品牌、知名特色品牌为重点，抓好19个省级农业标准化生产基地建设，确保“三品一标”新增认证产品50个、基地20万亩，争创国家级、省级农产品质量安全市和全国绿色食品原料基地。三是加快构建现代农业经营体系。加大龙头企业培植力度，确保新增规模以上企业20家。积极培育种养大户、家庭农场、农民合作社等新型经营主体，适度规模经营面积达到30%以上。抓好20个农产品批发市场完善提升，深化与北京首农集团的合作，使更多的农产品进入北京超市和社区，抢占高端市场。借助阿里巴巴等服务平台，扶持发展“淘宝村”。四是加快构建现代农业支撑体系。强化农业科技支撑，加快推进聊城国家农业科技园区建设，抓好5个农业科技孵化器、10个区域研发平台、100个新品种新技术展示基地建设；加快农业物联网等现代装备技术推广应用，每个县（市、区）至少新建成1处高标准示范园。实施“万名科技人员下乡”行动，培训新型农民20万人次以上。加强农业基础设施建设，实施金堤河流域、黄河滩区、沉沙池区治理，完成8座平原水库和输水管道主体工程，新增节水灌溉面积18万亩。

　　推进工业提档升级。加快工业向中高端迈进，确保规模以上工业增加值增长10%以上。一是加大传统产业改造力度。重点抓好有色金属、汽车及机械装备、化工等八大产业转型升级，推进信息化和工业化深度融合，实施信发集团年产30万吨高纯铝、泉林纸业250万吨秸秆综合利用等30个重点项目，确保技改投入增长12%以上。二是加大新兴产业培育力度。大力培育新材料、新能源、高端装备、节能环保等四大战略新兴产业，突出抓好新能源汽车、通讯光纤等重点产品，力争高新技术产业占比提高1.5个百分点以上。三是加大企业培植和项目建设力度。实施工业“双百”工程，集中力量培植100户重点企业，引导企业建立现代企业制度，完成10家企业上市材料申报，新增新三板挂牌企业20家以上；抓好100个重点工业项目建设，完成投资1000亿元。深入开展好企业工作日、金融工作日活动，积极帮助企业降低生产成本、化解担保圈风险，努力营造良好的发展环境。四是加大产业园区建设力度。着力抓好“5+3”千亿产业园区建设，推动企业集群式入园发展，力争主营业务收入达到3800亿元。突出抓好信发高新材料产业园建设，加快高端项目引进和产业配套，打造国际高新材料产业基地。五是加大过剩产能化解力度。对丧失自我修复能力的企业，通过兼并重组、债务重组等方式，实现市场出清，引导有限的资源流向更高效率的企业。启动化工产业转型升级攻坚战，用3年左右时间完成化工企业入园、转产或关停工作。

　　推进服务业跨越发展。调整优化传统业态，培育发展新兴业态，服务业占比提高2个百分点左右。一是实施现代物流业突破工程。抓好盖氏邦晔、千千佳等20家重点企业发展，改造提升烟店轴承市场等七个大型市场，加快六大百亿物流园区和16个重点项目建设，物流业增加值增长10%以上。二是实施电子商务示范带动工程。加快建设聊城电子商务产业园，培育创业大厦、当代国际大厦等电商集中区，推进电子商务进农村、进社区、进校园、进园区、进企业，创建国家电子商务示范城市。三是实施商贸业提升工程。支持传统商业创新经营业态，启动建设缤纷日月城、世纪天贸城等特大商业综合体，规划建设红星美凯龙等城市综合体，新建邮政便民服务站2500家。四是实施健康服务业培育工程。加快聊城大学医学院建设，打造聊城现代医疗综合体。鼓励社会资本投资医疗养老产业，促进鼎晖投资与市第三人民医院的合作，推动茌平千岛山庄、临清华美健康产业园等项目尽快投入使用，建设冀鲁豫三省交界健康医疗中心。五是实施文化旅游业壮大工程。高标准完成运河旅游发展及水生态体系规划编制，推进运河文化旅游产业带建设。整合古城区和东昌湖旅游资源，完成古城保护与改造，推动东昌湖风景区、阿胶养生文化苑创建5A级景区。加强地热资源保护和开发利用，积极发展大众化温泉旅游。以图书、演艺、动漫和非物质文化遗产展示为重点，扶持10个地方特色文化项目，培植壮大文化产业集群。六是实施金融高地建设工程。加快组建聊城商业银行，建成注册资本10亿元的大型融资性担保公司和聊城综合资产交易中心。引进商业银行等金融机构5家，银行机构存贷比力争提高2个百分点，普惠金融资产规模增长10%。同时，积极发展研发设计、商务咨询、信息技术、家政服务、休闲娱乐、体育健身等其他服务业，全面提升服务业发展的规模和水平。

　　（二）着力加大高效投入，确保项目建设实现新突破。深入实施大项目带动战略，全市固定资产投资达到2500亿元。一是狠抓精准招商。更加注重对接国家重点战略，注重围绕优势产业筛选项目，注重发挥商会、行业协会的桥梁纽带作用，注重发挥大企业的招商主体作用，突出招大引强，力争在引进重大项目、高科技项目、现代服务业项目上实现突破，确保全市招商引资到位资金800亿元以上、实际利用外资6300万美元以上。二是狠抓重点项目建设。突出抓好100个“三高一低”大项目建设，完善市级领导帮包、考核评比奖惩、第三方评估等机制，完成土地增减挂复垦面积8000亩、挖潜改造1万亩，开发未利用土地9000亩，确保项目顺利推进。三是狠抓交通基础设施建设。加快推进青兰高速、莘县至南乐高速建设，力争德商高速聊城至夏津段上半年通车；争取郑济高铁、聊泰铁路开工建设，聊城机场、京九高铁、聊邯长高铁、德郓高速等取得扎实进展。抓好干线公路建设，加强乡村道路改造，新建改建农村公路500公里。

　　（三）着力实施创新驱动，确保内生动力实现新增强。深入实施创新驱动和质量强市战略，努力提高全市核心竞争力。一是大力推进创新创业。推广“聊城创业大学+教学点”模式，发放创业担保贷款5亿元，抓好30个特色镇、600个专业村、31个小企业创业辅导基地建设，争取新登记各类市场主体5万户以上，争创国家级创业型城市。推进“智慧城市”建设，加快资源整合，基本建成云计算和大数据中心。二是大力推进平台建设。实施企业创新能力提升工程，支持企业组建产业技术创新联盟，力争新增省级以上研发平台11家，重点企业、重点行业实现省级以上技术创新平台全覆盖。加快创新孵化平台建设，培育科技成果研究推广中心10家。三是大力推进人才工作。研究出台高层次人才引进与管理办法，引进高层次专家200名以上、退休专家100名以上。加大人才国际化培养力度，开展海外高层次培训。积极发展职业教育，推动校企合作，新增高技能人才5500人以上。四是大力推进质量强市。全面加强标准、质量、品牌、信誉“四位一体”建设，积极参与国家和省标准制定，构建全市各领域标准体系，推进3个国家级质检中心、5个省级质检中心建设，抓好聊城大学质量学院、聊城质量发展研究中心的建设和发展。

　　（四）着力深化改革开放，确保发展空间实现新拓展。全面推进各领域改革。一是深化农村改革。落实集体所有权，积极稳妥开展农村集体产权制度改革，明确界定农民的集体成员权利；稳定农户承包权，依法将集体土地的承包经营权落实到每个农户，全面完成农村土地承包经营权确权颁证；放活土地经营权，完善县、乡、村土地流转体系，推动土地经营权、农民住房财产权抵押贷款试点，探索宅基地有偿使用和自愿退出机制，巩固完善农村基本经营制度。二是深化国有企业改革。完善国有资产监管体制，抓好国有资本投资运营公司建设，推动企业实施混合所有制改革，建立现代企业制度，完善法人治理结构。三是深化财税和投融资体制改革。完成“营改增”任务，完善国地税联合办税机制。对PPP项目实行财政贴息，力争市级股权引导基金总规模达到20亿元。加大地方政府债券、政策性银行长期低息贷款争取力度，切实保障民生项目建设。四是深化教育和医药卫生体制改革。大力推进名校办分校，鼓励发展民办教育，扩大办学自主权。推进分级诊疗、社会办医等9项改革，全面启动城市公立医院改革，加强区域医疗中心和乡村医疗机构建设，切实提高诊疗服务水平、减轻患者负担。

　　全方位扩大对外开放，培育对外开放新优势。一是千方百计扩大出口。以30户骨干企业为重点，建立“一对一”帮扶制度，积极组织企业参加“境外百展计划”，培植出口企业集群。二是多措并举稳定进口。支持信发、祥光等骨干企业发展，稳定、扩大资源性产品进口。鼓励企业开展新一轮技术改造，促进先进技术、关键设备和重要零部件进口。抓住中韩、中澳自贸协定正式生效的契机，积极推动日常消费品进口。三是加快建设“海外聊城”。依托全市骨干进口企业，抓好境外资源合作开发，建设资源性产业园区。推动新能源汽车、造纸等优势企业“走出去”，加快泉林纸业在美国项目建设，实现中通客车在台湾批量生产销售，支持乖宝宠物食品等企业建立海外生产基地和营销网络。四是切实优化服务环境。实行外经贸政策每月解读通报制度，加快市综合保税物流中心、宝信保税物流园、铁临物流保税仓库建设，做好临清与哈萨克斯坦物流专列推进工作，鼓励开展跨境电商业务。

　　（五）着力加强统筹联动，确保城乡面貌实现新变化。坚持以人的城镇化为核心，常住人口城镇化率达到48%，户籍人口城镇化率达到38.4%。一是切实提升城市规划水平。高标准完成城市总体规划、“聊茌东”都市区规划，做好与经济社会发展规划、土地利用总体规划的有机衔接。加强专项规划、控制性规划、片区规划和城市设计，突出生态景观，体现历史文化内涵和时代风貌。二是切实提升城市建设水平。积极推进湖河相连、水系进社区，规划建设金龙湖、凤凰苑公园，新增绿化面积200公顷。完善城市基础设施，抓好中心城区财干路等主次干道建设，新建垃圾处理厂3座；加强综合管廊和雨污分流管网建设，争创国家海绵城市试点市。加大回迁安置房建设力度，今年新开工2万户、竣工1.3万户，加快已开工项目建设进度，两年内全部完成回迁；建设公共租赁住房3000套，棚改货币化安置率达到50%。认真落实“去库存”任务，引导房地产企业多元化发展，向功能性地产转型。三是切实提升城市管理水平。按照“重心下移、属地管理、权责一致”原则，进一步理顺职责，构建“管理全覆盖、责任无盲区”的城管格局。大力推行精细化管理，加快数字化城管建设，开展违章建筑、占道经营、乱停乱放、三轮车违法营运等综合整治，确保市容市貌明显改善。四是切实提升城乡统筹水平。加快推进撤县设区、撤乡设镇，全面放宽城镇户籍限制，新增城镇户籍人口21万人。实施小城镇提升工程，完成第二批27个乡镇提升任务。扎实开展路域环境综合整治，加快推进交通、燃气、供热和污水处理设施向农村延伸，建成农村新型社区新居3000套。

　　（六）着力抓好节能减排，确保生态环境实现新改善。实行最严格的环境保护制度，有效遏制重污染天气频发态势。一是突出抓好大气污染防治。推动“工业绿动力”计划，抓好燃煤电厂、锅炉超低排放改造，对不达标企业依法实施限产停产。严控新增大气排放项目，完成鲁西集团一、二、四厂退城进园。推行煤炭清洁高效利用，严控燃煤质量标准，取缔燃煤小锅炉，推广新型采暖模式。深入开展扬尘综合整治，严肃查处秸秆焚烧行为。二是严格治理水污染。实施水污染防治行动计划，实现“千亩表流、百亩潜流”人工湿地县（市、区）全覆盖。加大污水处理厂建设力度，确保城市污水收集率达到90%以上，处理率、达标率达到100%。三是切实加强能源节约。开展百企节能低碳行动，实施节能重点项目86个，完成投资78亿元。支持发展循环经济，开展清洁生产对标活动，抓好工业固废综合利用。严格执行建筑节能标准，新增绿色建筑100万平方米，完成既有建筑节能改造30万平方米、太阳能光热建筑一体化80万平方米。四是大力推进生态保护。积极推进农业面源污染治理，推动秸秆综合利用，开展地下水超采区综合治理。广泛开展植树造林，新建国家级森林公园1处，全市林木绿化率达到40%以上。

　　（七）着力保障改善民生，确保群众生活实现新提高。牢固树立执政为民理念，不断加大民生投入，让广大群众享受更多改革发展成果。

　　全力打好精准脱贫攻坚战，确保完成15万人脱贫任务。一是建立强有力的工作机制。实行市级领导干部联系贫困乡镇、贫困村和市县部门帮包贫困村制度，逐级落实脱贫任务。动员社会各界积极参与，促进各类社会资源投入扶贫，凝聚脱贫攻坚整体合力。二是实施多种扶贫模式。广泛开展产业扶贫、就业扶贫、基础设施扶贫、金融扶贫、教育医疗扶贫，精选400家企业进村入户，发展“一村一品”特色产业，吸纳贫困户就业；彻底解决贫困群众吃水困难；实行集中建设、集中服务，用两年时间完成贫困家庭危房改造；对贫困家庭学前适龄儿童免收保教费，普通高中生免除学杂费，大中专和中高职学生每年补助3000元。三是强化社会保障“兜底”作用。对丧失劳动能力的贫困人口，全部纳入低保体系，今年县（市、区）低保线要达到国家扶贫标准线，2018年达到省定扶贫标准线。

　　认真办好民生实事。围绕关系群众切身利益的问题，集中力量办好十大民生实事。一是开展免费就业培训。安排市级创业带动就业扶持资金3000万元，开展职业技能和创业培训3.5万人，确保新增城镇就业4.5万人。二是推动社会保障扩面提质。建立职工大病保险制度，开展职工长期护理保险。新增养老床位3500张，建设社区日间照料中心9处、农村幸福院120处。困境儿童基本生活费标准提高到每人每月720元。三是推进教育均衡发展。新建、改扩建公办幼儿园153所，新建中小学校56所，市实验幼儿园建成招生，开工建设聊城一中新校区，加强中小学食堂建设和管理。四是实施卫生计生“五项免费”工程。实施婚前医学免费检查、新生儿疾病免费筛查、孕期胎儿无创基因免费检测、农村贫困家庭儿童先心病免费救治和农村妇女“两癌”免费检查。五是开展公益性文化服务。启动市图书馆、档案馆、方志馆文化综合体建设，60%以上的行政村建成综合性文化服务中心；举办公益演出2000场，免费放映电影6万场。六是推进老旧小区和农村危房改造。改造城镇老旧小区100个、农村危房4800户，完成农村无害化卫生厕所改造22万户，实施农村供暖改造。七是实施全民健身工程。建设体育健身工程500个，培训社会体育指导员1000人，举行全民健身运动会。八是改善中心城区交通条件。新增新能源公交车220辆、改造候车设施70个；建设城市公共自行车出行系统。开展道路微循环改造，规划建设过街天桥，新增停车位2000个，有效缓解交通拥堵。九是强化食品药品监管。实施食品药品“智慧监管”工程，食品抽检批次增长20%，评价性抽检合格率保持95%以上。十是加强基层法律服务。实施法律服务“一乡镇一团队、一村居一顾问”工程，形成城镇半小时、农村一小时公共法律服务圈。

　　改革创新社会治理。全面推进社会主义核心价值体系建设，深入开展群众性精神文明创建活动；充分利用好水城明珠大剧场等文化设施，丰富群众精神文化生活。推进基层民主自治规范化建设，加快建立多元参与、多方共治的社区治理机制。推动军民融合，力争双拥创城“四连冠”。认真落实全面二孩政策，提高出生人口素质，促进人口均衡发展。深化平安聊城建设，完善社会治安立体化防控体系，建设交通安全样板市。加强公共基础消防设施建设，完成103个乡镇消防站建设，形成覆盖城乡的灭火救援体系。牢牢守住安全底线，健全完善安全生产责任、隐患排查治理、预防控制、应急救援等四大体系。扎实做好民族宗教、外事侨务、台湾事务、防震减灾、妇女儿童、老龄、人防、油区、气象等工作，确保社会和谐稳定。

　　四、切实加强和改进政府自身建设

　　各位代表，实施好“十三五”规划，全面完成今年经济社会发展各项目标任务，对政府自身建设提出了新的更高要求。我们一定坚持从严治政、改革创新，加快建设法治政府、创新政府、责任政府、服务政府和廉洁政府。

　　(一)切实做到依法行政，加快建设法治政府。坚持在党的领导下、在法治轨道上开展工作，自觉运用法治思维和法治方式深化改革、推动发展、化解矛盾、维护稳定。加强与人大沟通协调，认真做好地方立法工作。严格执行政府议事规则和重大决策程序规定，切实发挥好重大决策咨询委员会、法律顾问委员会和第三方评估机构的作用。进一步健全政府信息公开制度，完善社会监督和舆论监督机制。大力推进服务型执法，加强执法监督，规范执法行为。自觉接受人大及其常委会的监督，主动加强同人民政协的民主协商，认真做好人大议案、代表建议和政协提案办理工作。

　　(二)切实做到求真务实，加快建设创新政府。加强对政府工作人员的培训力度，引导广大干部适应新常态、学习新知识、增长新才干。深入推进简政放权，削减市级行政审批29项。加强电子政务建设，推进行政审批联审联办、网上办理、限时办结。完善中介服务超市，每类行政审批中介服务机构均在3家以上。创新行政管理，最大限度地采用购买服务的方式提供基本公共服务。建立流程化管理体制机制，对事关全局的重点工作实行流程化管理，形成决策部署、执行落实、督导检查、办理反馈的完整链条，确保各项工作落到实处、取得实效。

　　(三)切实做到担当尽责，加快建设责任政府。强化政治意识、大局意识、核心意识、看齐意识，做到勇于负责、敢于担当。完善考核评价体系，旗帜鲜明地给干事创业的干部撑腰鼓劲，理直气壮地为敢于担当者而担当，对主动作为、做有成效的予以表彰，对不作为的部门和干部严肃问责，让敢于担当的人安心、放心，让拼搏实干的人宽心、舒心。建立合理的容错机制、及时的纠错机制和正常的免责制度，营造宽容失败、鼓励担当的环境和氛围。

　　(四)切实做到执政为民，加快建设服务政府。巩固拓展党的群众路线教育实践活动成果，健全完善“三严三实”作风建设长效机制。坚持深入基层、深入群众，保持同群众的密切联系。加强调查研究，做好人民建议征集工作，使各项工作更加符合群众意愿，得到社会各界的理解和支持。尊重、关心、保护企业家，切实为企业发展搞好服务。扎实开展群众工作日活动，做好群众来信来访、市长公开电话等工作，及时解决群众反映的困难和问题，不断增强广大群众的满意度和幸福感。

　　(五)切实做到从严治政，加快建设廉洁政府。坚决把党的纪律和规矩挺在前面，严格落实党风廉政建设责任制，不断健全惩治和预防腐败体系。严格遵守中央和省委、市委各项规定，完成公务用车改革，杜绝公车私用。加快市公共资源交易中心运营，严格执行公共资金使用透明化等制度，切实做到以制度管人管事管钱。全面推进预算绩效管理和“三公”经费、财政预算公开，完善落实国库集中支付和公务卡制度，对公共资金、国有资产、国有资源和领导干部履行经济责任情况实行审计全覆盖。坚持有腐必反、有贪必肃，加大监督检查力度，对违纪违规行为发现一起、查处一起，真正营造风清气正的良好环境。

　　各位代表，全面小康号角起，千帆竞发战鼓催。让我们紧密团结在以习近平同志为总书记的党中央周围，在省委、省政府和市委的坚强领导下，齐心协力，扎实苦干，坚决实现“十三五”良好开局，为全面建成小康社会、创造聊城人民更加美好的幸福生活而努力奋斗！
